# Risk-enhancing factors and social determinants of health in risk assessment for atherosclerotic cardiovascular disease

**DOI:** 10.1371/journal.pone.0312756

**Published:** 2024-10-25

**Authors:** Yiyi Zhang, Jaejin An, Mengying Xia, Hui Zhou, Yifei Sun, Joanie Chung, Mengnan Zhou, Soon Kyu Choi, Kerresa L. Morrissette, Paul Muntner, Monika M. Safford, Carmen R. Isasi, Alka M. Kanaya, Brandon K. Bellows, Lisandro D. Colantonio, Kristi Reynolds, Andrew E. Moran

**Affiliations:** 1 Division of General Medicine, Columbia University Irving Medical Center, New York, NY, United States of America; 2 Department of Research & Evaluation, Kaiser Permanente Southern California, Pasadena, CA, United States of America; 3 Department of Health Systems Science, Kaiser Permanente Bernard J. Tyson School of Medicine, Pasadena, CA, United States of America; 4 Department of Biostatistics, Mailman School of Public Health, Columbia University Irving Medical Center, New York, NY, United States of America; 5 Department of Epidemiology, University of Alabama at Birmingham, Birmingham, AL, United States of America; 6 Division of General Internal Medicine, Department of Medicine, Weill Cornell Medicine, New York, NY, United States of America; 7 Department of Epidemiology and Population Health, Albert Einstein College of Medicine, Bronx, NY, United States of America; 8 Department of Medicine, University of California San Francisco, San Francisco, CA, United States of America; Instituto Nacional de Cardiologia Ignacio Chavez, MEXICO

## Abstract

**Background:**

The Pooled Cohort Equations (PCEs) do not accurately estimate atherosclerotic cardiovascular disease (ASCVD) risk in certain populations. The 2018 AHA/ACC cholesterol guideline identified risk-enhancing factors as a supplement to PCEs-based risk assessment. However, the role of each risk-enhancing factor in ASCVD risk assessment has not been well quantified. Further, social determinants of health (SDOH) are not included in the PCEs nor considered as risk-enhancing factors in the US cholesterol guideline. We sought to evaluate ASCVD risk associated with each risk-enhancing factor and commonly collected SDOH including education, income, and employment status, and to assess if adding risk-enhancing factors and SDOH to the PCEs improve ASCVD risk prediction.

**Methods:**

We included individuals aged 40 to 75 years, without ASCVD or diabetes at baseline, and with low-density lipoprotein cholesterol 70–189 mg/dL from two contemporary prospective cohort studies (MESA and REGARDS) and from Kaiser Permanente Southern California (KPSC). The primary endpoint was incident ASCVD defined as nonfatal myocardial infarction, fatal coronary heart disease, or fatal or nonfatal stroke over a 10-year period (median follow-up 10 years). We used Cox proportional hazards models to estimate associations between risk-enhancing factors and SDOH with ASCVD. We also assessed changes in model performance after adding risk-enhancing factors and SDOH to the PCEs.

**Results:**

We included 13,863 adults (mean age 60.7 years) from the prospective cohorts and 307,931 adults (mean age 54.8 years) from KPSC. Risk-enhancing factors including hypercholesterolemia, hypertriglyceridemia, metabolic syndrome, and chronic kidney disease were associated with a higher ASCVD risk, independent of 10-year risk estimated by the PCEs. Low education, low income, and unemployment were also associated with higher ASCVD risk. While adding individual risk-enhancing factors or SDOH to the PCEs had limited impact on model performance, adding multiple risk-enhancing factors and SDOH simultaneously led to modest improvements in discrimination (C-index increased by up to 0.07), calibration (integrated Brier score reduced by up to 2.3%), and net reclassification improvement up to 41.4%.

**Conclusions:**

These findings suggest including SDOH and risk-enhancing factors may improve ASCVD risk assessment.

## Introduction

The 2013 American Heart Association (AHA) and American College of Cardiology (ACC) guideline on the management of cholesterol recommended using the Pooled Cohort Equations (PCEs) to estimate 10-year atherosclerotic cardiovascular disease (ASCVD) risk to guide statin therapy [1]. However, validation studies have shown that PCEs may overestimate ASCVD risk by 60–90% in some US cohorts and underestimate risk in certain racial/ethnic groups or those with lower socioeconomic status [2–8]. Acknowledging the limitations of the PCEs, the 2018 AHA/ACC cholesterol guideline identified risk-enhancing factors that should be considered in ASCVD risk assessment, particularly among those with borderline or intermediate 10-year risk [4]. The presence of one or more risk-enhancing factors can be used to confirm a higher risk state and thereby supports a decision to initiate or intensify statin therapy [4]. However, the guideline did not quantify how much a risk-enhancing factor may change the 10-year predicted ASCVD risk for an individual patient, making decisions to treat or not to treat, informed by the risk-enhancing factors, somewhat subjective. Each risk-enhancing factor may confer different risk for ASCVD beyond that predicted by the PCEs. For example, the presence of chronic kidney disease (CKD) may lead to a larger increase in 10-year risk than metabolic syndrome [9].

Additionally, social determinants of health (SDOH) are important risk factors for ASCVD but are not included in the PCEs nor as risk-enhancing factors in the current guideline [10, 11]. Studies have shown that the PCEs underestimate ASCVD risk in individuals with social deprivation or from disadvantaged neighborhoods [12, 13], which may lead to underuse of statins and exacerbation of cardiovascular health disparities. Although area-level social deprivation index was considered as an optional predictor in the recently developed Predicting Risk of CVD Events (PREVENT) equations, the role of individual-level SDOH were not assessed in PREVENT [14]. Additionally, PREVENT did not examine the impact of other risk-enhancing factors except for estimated glomerular filtration rate [14].

This study sought to (1) quantify ASCVD risk associated with each risk-enhancing factor recommended in the 2018 AHA/ACC cholesterol guideline and with commonly collected SDOH including education, income, and employment status, beyond the risk estimated by the PCEs; and (2) assess if adding individual or combination of risk-enhancing factors and SDOH to the PCEs improve ASCVD risk prediction. We analyzed data from two contemporary prospective cohort studies as well as from Kaiser Permanente Southern California (KPSC), a large US integrated healthcare system serving a diverse population.

## Methods

### Study design and cohorts

The present analysis was based on two complementary data sources: (1) pooled data from two large prospective cohort studies: Multi-Ethnic Study of Atherosclerosis (MESA) [15] and REasons for Geographic And Racial Disparities in Stroke (REGARDS) [16], and (2) electronic health records from KPSC. We chose to include these specific cohorts instead of other older cardiovascular cohort studies because ASCVD event rates were much higher in older cohorts and may not reflect ASCVD risk in the contemporary US population [2]. Details of the design of each study are reported in **Supplemental Methods in S1 File**. Data from the two cohort studies were collected from January 2000 to December 2019; electronic health records from KPSC were collected from September 30, 2009 to December 31, 2019, and were accessed and pulled for this analysis on January 10, 2023. Study protocols were approved by the Institutional Review Boards at Columbia University and at KPSC. All participants in the pooled cohort provided written informed consent, and informed consent for the KPSC population was waived given the retrospective nature of the study.

The current analysis was restricted to non-Hispanic White and non-Hispanic Black individuals from the pooled cohort and KPSC who were 40 to 75 years of age, without existing ASCVD or diabetes at baseline, and with low-density lipoprotein cholesterol 70–189 mg/dL (**S1 Fig in S1 File**). These individuals would be recommended by the 2018 AHA/ACC cholesterol guideline to have their 10-year ASCVD risk calculated by the PCEs in order to guide statin initiation [4]. We excluded participants who were already taking a statin at baseline or had missing data to calculate 10-year ASCVD risk.

### Risk-enhancing factors, SDOH, and clinical data collection

This study analyzed eight risk-enhancing factors recommended in the 2018 AHA/ACC cholesterol guideline (including family history of premature ASCVD, hypercholesterolemia, hypertriglyceridemia, metabolic syndrome, CKD, female conditions, elevated high-sensitivity C-reactive protein [hsCRP], and chronic inflammatory conditions), as well as nine individual-level SDOH (including low education, low household income, unemployment, marital status) and area-level SDOH (neighborhood low education, neighborhood low household income, neighborhood high unemployment rate, neighborhood high poverty, and neighborhood deprivation index) that were not yet included in the cholesterol guideline. Details of the definitions of each risk enhancing factor and SDOH are reported in **S1 Table in S1 File**. In the pooled cohort, demographic characteristics, lipids, and other cardiovascular risk factors were assessed following standardized protocols in each study [15, 16]. Race and ethnicity, family history of premature ASCVD, education, annual household income, employment status, and female conditions (including premature menopause, gestational diabetes, gestational hypertension) were based on participants’ self-report. In KPSC, race and ethnicity was based on a combination of self-report and administrative data, and risk-enhancing factors such as CKD and chronic inflammatory conditions were identified through electronic health records using validated algorithms and clinical disease management registries [17, 18]. In both the pooled cohort and KPSC, area-level SDOH were derived by linking participants’ addresses via geocoding to US Census tract data [19, 20]. We also calculated a summary neighborhood deprivation index using the algorithm developed by the Agency for Healthcare Research and Quality (AHRQ) based on seven area-level indicators of poverty, education, employment, and physical environment, with a lower value indicative of greater neighborhood deprivation [21].

### ASCVD events and study follow-up

The primary endpoint was incident ASCVD defined as nonfatal myocardial infarction (MI), fatal coronary heart disease (CHD), or fatal or nonfatal stroke over a 10-year period. For the pooled cohort, events were ascertained and adjudicated within each study following a specific protocol [15, 16]. The diagnosis of an MI required at least two of the following: symptoms indicative of ischemia, electrocardiographic or other imaging abnormalities consistent with MI, and a rising and/or falling pattern of cardiac biomarkers over at least 6 hours with a peak above the upper limit of normal. The diagnosis of stroke required a persistent central neurologic deficit lasting >24 hours and/or brain imaging consistent with acute stroke. For KPSC, we used previously validated algorithms and shown high positive predictive values (>90%) to identify MI and stroke [22–24]. Specifically, MI was identified by principal hospital discharge diagnoses with International Classification of Diseases Ninth Revision (ICD-9) codes 410.x0, 410.x1 and ICD-10 codes I21.x, [22] and stroke by principal hospital discharge diagnoses with ICD-9 433.x1, 434.x1, 436.xx, and ICD-10 I63.x, G46.3, G46.4. [23, 24] Deaths from CHD and stroke were identified using ICD-10 (I20-I25, I60-I69) from membership files, hospital records, and death files from state sources [25]. Additionally, we performed sensitivity analysis using both principal and secondary hospital discharge diagnosis codes to define MI events since some previous studies found that using only principal diagnosis may underestimate the incidence of MI events [26].

Eligible individuals were followed from study entry (defined as the first eligible visit in the pooled cohort and 09/30/2009 in KPSC) for 10 years for an incident ASCVD event. Follow-up time was censored at the incident ASCVD event, death, statin initiation, end of study, loss to follow-up, or disenrollment (for KPSC members), whichever occurred first.

### Statistical analysis

All analyses were performed in the pooled cohort and in KPSC separately. Individual characteristics at baseline were described for the pooled cohort and for KPSC. Since the PCEs are known to overestimate risk in some of the cohorts and underestimate risk in others [6, 27], we recalibrated the PCEs to the pooled cohort and KPSC data, respectively, to avoid under- or over-estimating the contribution of risk-enhancing factors and SDOH in modifying the PCEs-based risk estimates [28]. During recalibration, the baseline survival function and mean value of risk factors in the original PCEs were replaced with sex- and race-specific values (White women, White men, Black women, Black men) estimated from the pooled cohort and KPSC, respectively [28] (**S2 Table in S1 File**). We assessed mean calibration as the ratio of predicted to observed event rates. The calibrated PCEs (cPCEs) were then used in all subsequent analyses.

To assess the association between risk-enhancing factors and SDOH with ASCVD beyond risks estimated by the cPCEs, we fitted separate Cox proportional hazards model including individual risk-enhancing factors and SDOH as the independent variables, adjusting for the 10-year risk estimated by the cPCEs. The proportional hazards assumption was checked by plotting the log(-log(survival)) vs. log(survival time) and by using Schoenfeld Residuals. Additionally, we estimated the population attributable fraction (PAF) to quantify the proportion of 10-year ASCVD events attributable to each risk-enhancing factor and SDOH by fitting separate models for individual risk-enhancing factors and SDOH and adjusting for the cPCEs [29].

To assess if incorporating risk-enhancing factors and SDOH into 10-year ASCVD risk assessment improves prediction accuracy beyond cPCEs, we fitted separate Cox models with (1) cPCEs only and (2) cPCEs plus individual or multiple risk-enhancing factors and SDOH. To avoid potential overfitting, risk-enhancing factors and SDOH with high correlation (>0.7) were not included simultaneously. Then, we estimated the change in discrimination (estimated by Harrell’s C-index) and calibration (estimated by integrated Brier score [IBS]) between the two models. We used nonparametric bootstrapping to estimate the 95% CIs for the difference in Harrell’s C-index and percent change in IBS. Additionally, we calculated categorical net reclassification improvement (NRI) for ASCVD events and non-events to assess how each risk-enhancing factor may reclassify individuals into higher or lower ASCVD risk categories. We used cut-points at 10-year risk of 5%, 7.5%, and 20% to define risk categories as recommended in the 2018 AHA/ACC cholesterol guideline [4]. A 2-sided P < 0.05 determined statistical significance. Analyses were performed using R version 4.0.2 (Vienna, Austria) and SAS version 9.4 (SAS Institute Inc, Cary, North Carolina).

## Results

### Prevalence of risk-enhancing factors

We included 13,863 participants from the pooled cohort (2,898 from MESA and 10,965 from the REGARDS study) and 307,931 participants from KPSC (**Table 1**). Mean (SD) age was 60.7 (7.8) years in the pooled cohort and 54.8 (9.0) years in KPSC. In the pooled cohort, 58.6% were women, 61.4% self-identified as non-Hispanic White, 34.8% had ≥3 risk-enhancing factors out of a total of 7 risk-enhancing factors evaluated in the pooled cohort according to definitions in the 2018 AHA/ACC cholesterol guideline, and 47.2% had ≥3 adverse SDOH out of 9 evaluated (**Table 2)**. In KPSC, 59.3% were women, 80.4% were non-Hispanic White, 1.4% had ≥3 risk-enhancing factors out of 7 evaluated in KPSC, and 9.7% had ≥3 adverse SDOH out of 5 evaluated.

**Table 1:** Baseline characteristics of study population.

	Pooled Cohort (N = 13,863)	KPSC (N = 307,931)
Age, years	60.7 (7.8)	54.8 (9.0)
Year of enrollment	2000–2007	2009
Sex		
Female	8,117 (58.6%)	182,515 (59.3%)
Male	5,746 (41.4%)	125,416 (40.7%)
Race		
Non-Hispanic Black	5,356 (38.6%)	60,423 (19.6%)
Non-Hispanic White	8,507 (61.4%)	247,508 (80.4%)
Smoking status		
Never	6,669 (48.1%)	211,670 (68.7%)
Former	5,048 (36.4%)	73,918 (24.0%)
Current	2,110 (15.2%)	22,343 (7.3%)
Body mass index, kg/m^2^	28.6 (5.9)	29.0 (6.3)
Lipids, mg/dL		
Total cholesterol	200 (31)	203 (32)
HDL cholesterol	54 (17)	55 (15)
LDL cholesterol	123 (27)	124 (26)
Triglycerides	102 (75, 143)	102 (74, 145)
Blood pressure, mm Hg		
Systolic	125 (17)	124 (14)
Diastolic	76 (10)	74 (10)
hsCRP, mg/L	2.1 (0.9, 4.7)	2.0 (0.8, 5.1)
Anti-hypertensive medication use	4,698 (33.9%)	104,291 (33.9%)
Female conditions		
Gestational diabetes	25 (0.3%)	2,153 (1.2%)
Gestational hypertension	53 (0.7%)	946 (0.5%)
Preeclampsia	NA	1,916 (1.0%)
Premature menopause	1,248 (15.4%)	246 (0.1%)

* Values are mean (SD), median (25th, 75th percentile), or number (%).

HDL: high-density lipoprotein; hsCRP: high-sensitivity C-reactive protein; KPSC: Kaiser Permanente Southern California; LDL: low-density lipoprotein; NA: not available.

**Table 2:** Prevalence of risk-enhancing factors and social determinants of health (SDOH).

	Pooled Cohort (N = 13,863)	KPSC (N = 307,931)
**Risk-enhancing factors according to the 2018 AHA/ACC cholesterol guideline**		
Family history of premature ASCVD	2131 (15.4%)	NA
Hypercholesterolemia	1797 (13.0%)	18736 (6.1%)
Metabolic syndrome	3805 (27.4%)	44926 (14.6%)
Chronic kidney disease	869 (6.3%)	11042 (3.6%)
Chronic inflammatory conditions	NA	6448 (2.1%)
Hypertriglyceridemia	2040 (14.7%)	32766 (10.6%)
Elevated hsCRP	6921 (49.9%)	2497 (0.8%) †
Female conditions (premature menopause, gestational diabetes, gestational hypertension, preeclampsia) ‡	1315 (16.2%)	4746 (2.6%)
Number of risk-enhancing factors	2.2 (1.2)	0.4 (0.7)
0	263 (2.8%)	217441 (70.7%)
1–2	5757 (62.4%)	86062 (27.9%)
≥3	3213 (34.8%)	4428 (1.4%)
**Adverse SDOH**		
Less than high school education	1008 (7.3%)	NA
Low household income	7205 (52.0%)	NA
Unemployed	341 (2.5%)	NA
Not married or living as married	5240 (37.8%)	NA
Low neighborhood education	5288 (38.1%)	49596 (16.1%)
Low neighborhood household income	10146 (73.2%)	60493 (19.6%)
High neighborhood poverty	2964 (21.4%)	11510 (3.7%)
High neighborhood unemployment	3391 (24.5%)	19157 (6.2%)
AHRQ neighborhood deprivation index §	50.7 (46.8, 54.9)	56.9 (55.2, 58.9)
Number of adverse SDOH	2.7 (2.1)	0.7 (1.1)
0	1610 (14.1%)	208083 (67.6%)
1–2	4413 (38.7%)	70130 (22.7%)
≥3	5390 (47.2%)	29718 (9.7%)

* Values are mean (SD), number (%), or median (25^th^, 75^th^ percentiles).

† hcCRP measurement was missing for 98.4% of the KPSC cohort.

‡ Prevalence of female condition was calculated among women only.

§ Bottom quintile of AHRQ neighborhood deprivation index was considered an adverse SDOH in this study.

ASCVD: atherosclerotic cardiovascular disease; hsCRP: high-sensitivity C-reactive protein; KPSC: Kaiser Permanente Southern California; NA: not available.

### Performance of the original and recalibrated PCEs

During a median follow-up of 10 years, 785 (5.7%) ASCVD events occurred in the pooled cohort and 7,165 occurred in KPSC (2.3%). In the pooled cohort, the original PCEs overestimated 10-year ASCVD risk by 22% in White women to 48% in Black men (**S3 Table in S1 File**). In KPSC, the original PCEs overestimated 10-year risk by 68% in White women to 125% in Black men. In sensitivity analysis using both principal and secondary diagnoses codes to identify MI events in KPSC, the original PCEs overestimated 10-year risk by up to 106%. In both the pooled cohort and KPSC, mean predicted 10-year risks were closer to observed 10-year risks in all sex/race groups after recalibration except for White women. The Harrell’s C-index for the original PCEs ranged from 0.65 to 0.73 in the pooled cohort, and from 0.69 to 0.74 in KPSC (**S4 Table in S1 File**). The C-indexes were unchanged after recalibration.

### Associations between risk-enhancing factors and incident ASCVD

**Fig 1:**
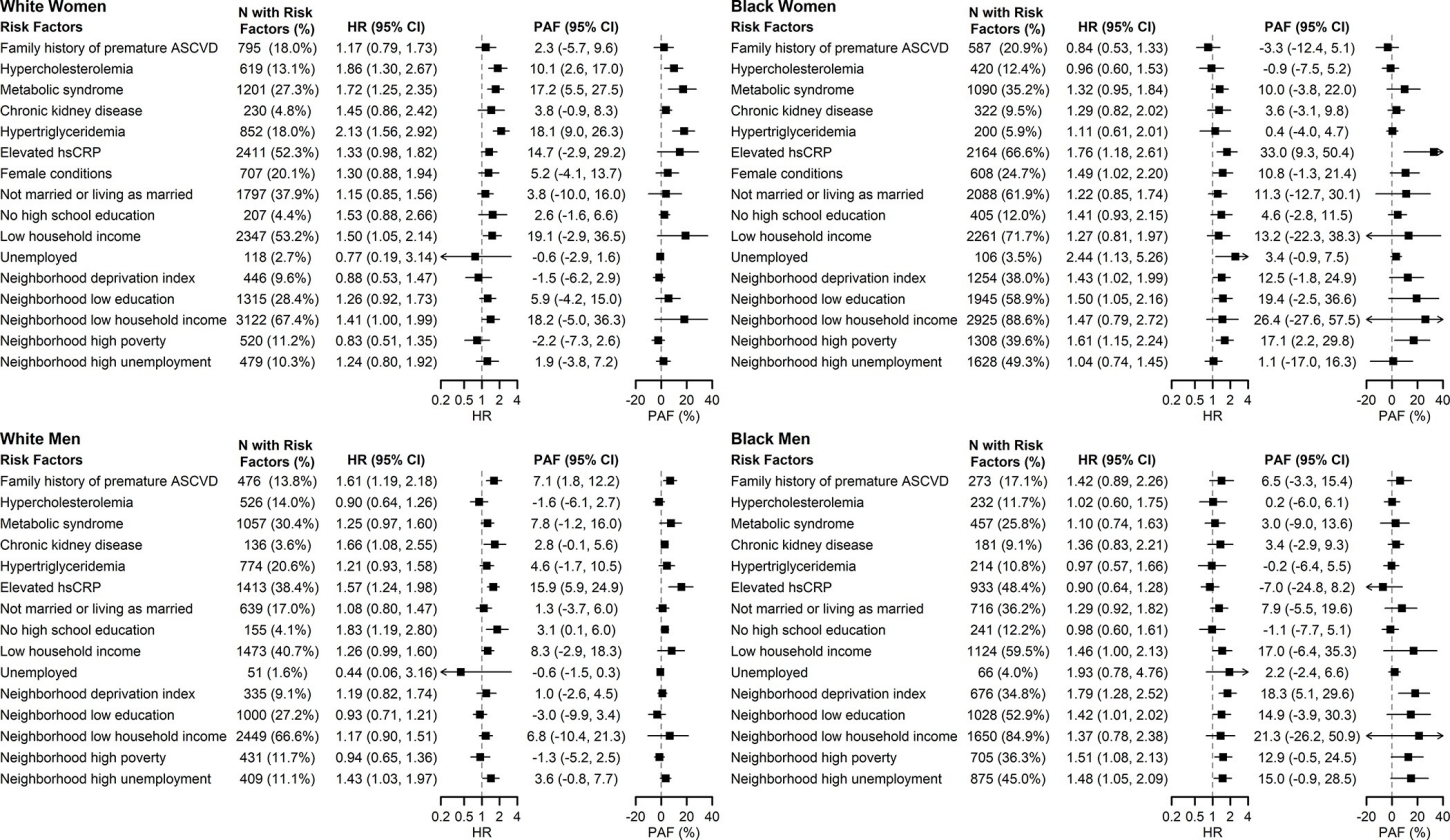
Adjusted hazard ratios and population attributable fractions of ASCVD associated with each risk-enhancing factor and social determinants of health, pooled cohort. Caption: AHRQ: Agency for Healthcare Research and Quality; ASCVD: atherosclerotic cardiovascular disease; HR: hazard ratio; PAF: population attributable fraction.

In the pooled cohort, family history of premature ASCVD, hypercholesterolemia, metabolic syndrome, CKD, hypertriglyceridemia, elevated hsCRP, and female conditions were associated with ASCVD in some of the sex/race groups but not others, after adjusting for 10-year risk estimated by the cPCEs (**Fig 1**). Individual-level and area-level SDOH of low education, low income, and unemployment were also associated with ASCVD, particularly in non-Hispanic Black participants (hazard ratios [HR] ranged from 1.41 to 2.44). Hypertriglyceridemia had the highest PAF in White women (PAF = 18.1%, 95% CI: 9.0%-26.3%), hsCRP had the highest PAF in White men (PAF = 15.9%, 95% CI: 5.9%-24.9%) and Black women (PAF = 33.0%, 95% CI: 9.3%-50.4%), and AHRQ neighborhood deprivation index had the highest PAF in Black men (PAF = 18.3%, 95% CI: 5.1%-29.6%).

**Fig 2:**
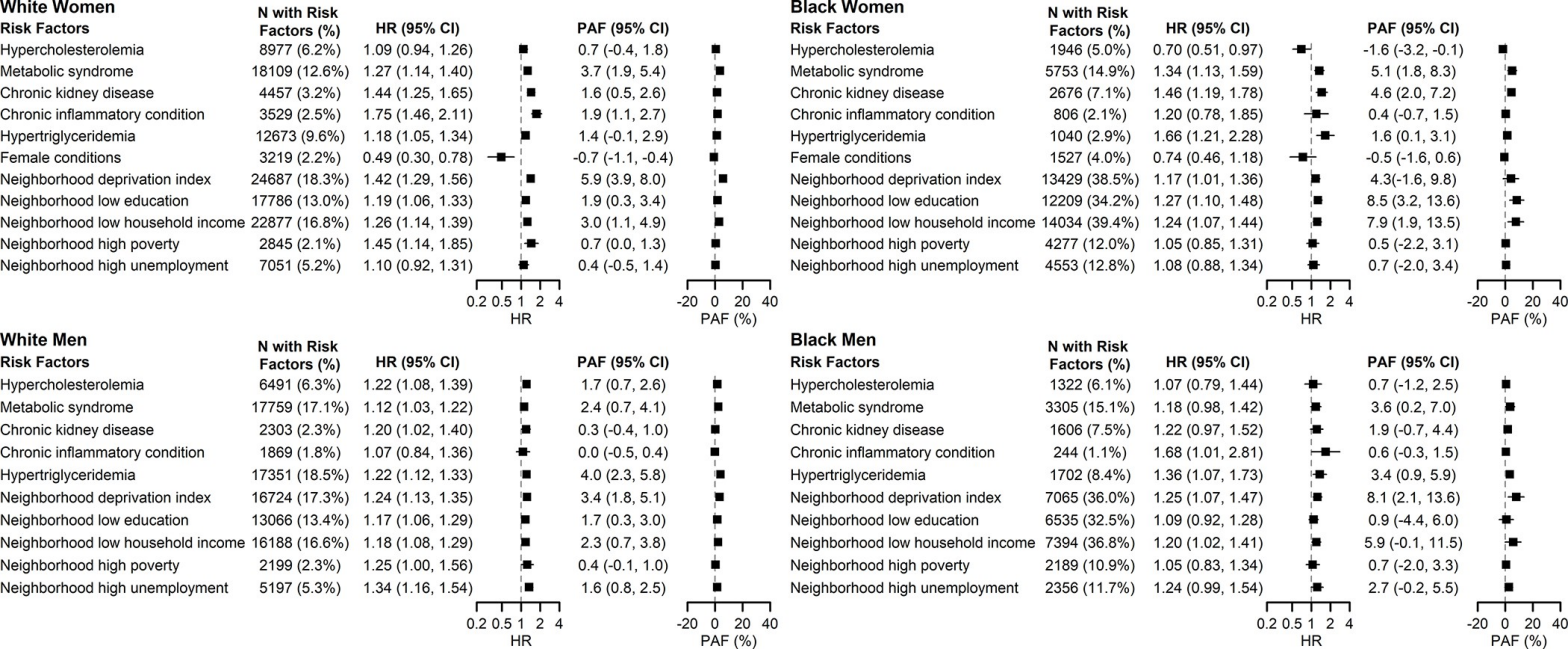
Adjusted hazard ratios and population attributable fractions of ASCVD associated with each risk-enhancing factor and social determinants of health, KPSC. Caption: AHRQ: Agency for Healthcare Research and Quality; ASCVD: atherosclerotic cardiovascular disease; HR: hazard ratio; KPSC: Kaiser Permanente Southern California; PAF: population attributable fraction.

In KPSC, hypertriglyceridemia, AHRQ neighborhood deprivation index, and neighborhood household income were associated with incident ASCVD in all four sex/race groups (**Fig 2** and **S5 Table in S1 File**). Hypercholesterolemia, metabolic syndrome, CKD, chronic inflammatory conditions, and area-level SDOH including neighborhood education, poverty, and unemployment were associated with ASCVD in some of the sex/race groups but not others. In sensitivity analysis using both principal and secondary diagnosis codes to identify MI events, the same risk enhancing factors were identified to be associated with incident ASCVD. The PAF for AHRQ neighborhood deprivation index ranged from 3.4% (95% CI: 1.8%-5.1%) in White men to 8.1% (95% CI: 2.1%-13.6%) in Black men.

### Change in prediction performance after adding risk-enhancing factors to the cPCEs

**Fig 3:**
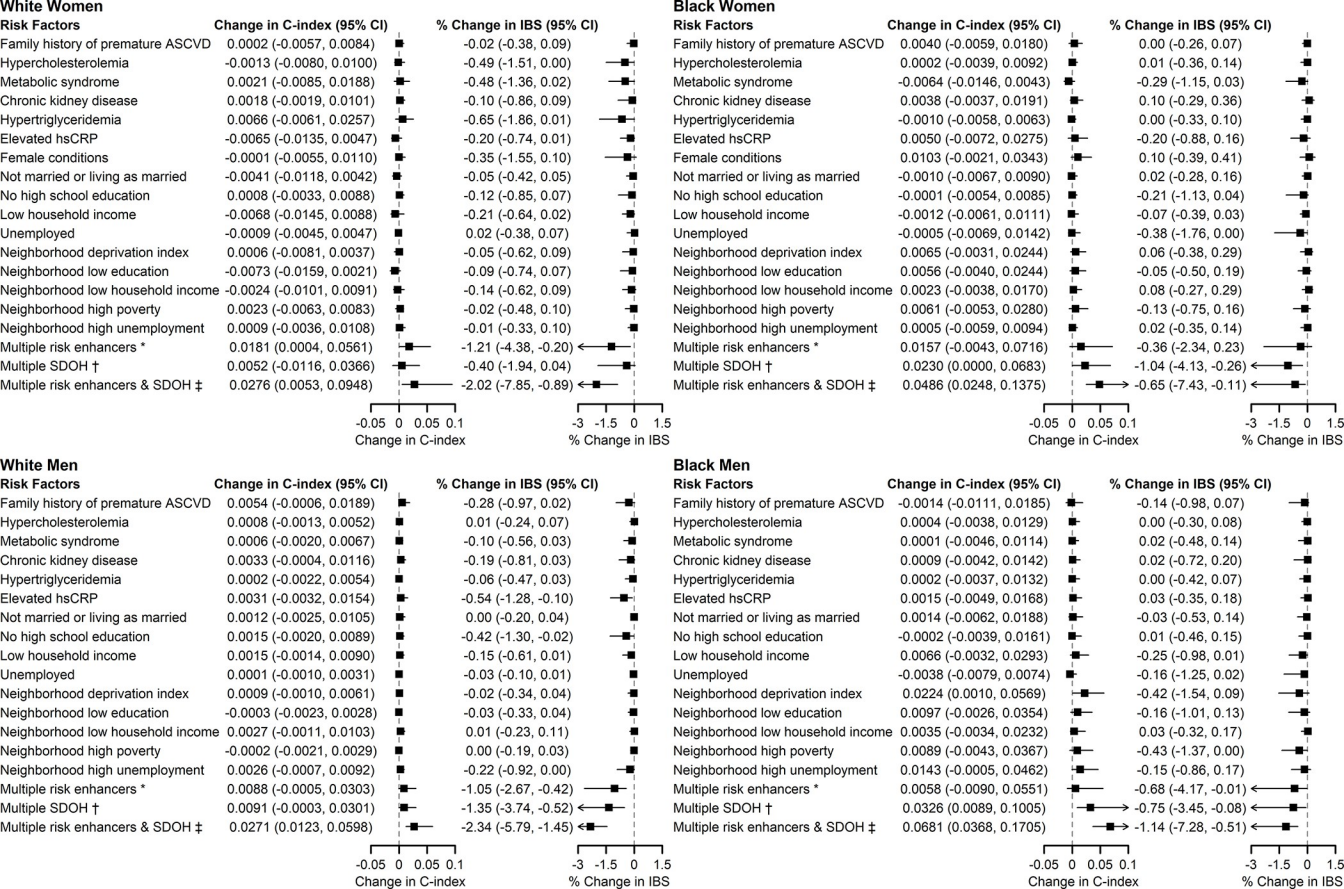
Differences in Harrell’s C-index and percent change in integrated Brier score comparing risk models with and without individual or combination of risk-enhancing factors and social determinants of health, pooled cohort. Caption: * Multiple risk enhancers include family history of premature ASCVD, hypercholesterolemia, metabolic syndrome, CKD, hypertriglyceridemia, elevated hsCRP, and female conditions (in women only). † Multiple SDOH include no high school education, low household income, unemployment, neighborhood deprivation index, neighborhood low education, neighborhood low household income, neighborhood high poverty, neighborhood high unemployment. ‡ Multiple risk enhancers & SDOH include all the above. AHRQ: Agency for Healthcare Research and Quality; ASCVD: atherosclerotic cardiovascular disease; IBS: integrated Brier score.

In the pooled cohort, adding individual risk-enhancing factors to risk prediction did not improve the C-index compared to models with cPCE only, except for AHRQ neighborhood deprivation index in Black men (ΔC-index = 0.0224, 95% CI: 0.0010–0.0569; **Fig 3**). Additionally, adding hypercholesterolemia, hsCRP, low education, unemployment, and neighborhood poverty to risk prediction led to modest improvements in IBS in some sex/race groups but did not statistically improve C-index. Further, when adding to the risk prediction models simultaneously all risk-enhancing factors and SDOH that were associated with ASCVD in at least one of the sex/race groups, there were statistically significant improvements in the C-index and IBS in all sex/race groups (ΔC-index ranged from 0.0271 in White men to 0.0681 in Black men; % change in IBS ranged from -0.65% in Black women to -2.34% in White men). Similarly, adding individual risk-enhancing factors or SDOH to the cPCEs did not improve reclassification, whereas adding multiple risk-enhancing factors and SDOH simultaneously (i.e., all risk-enhancing factors and SDOH that were associated with ASCVD in at least one of the sex/race groups) was associated with the greatest number of individuals correctly reclassified (overall NRI ranged from 1.4% in White women to 41.1% in Black men; **S2 Fig in S1 File**).

**Fig 4:**
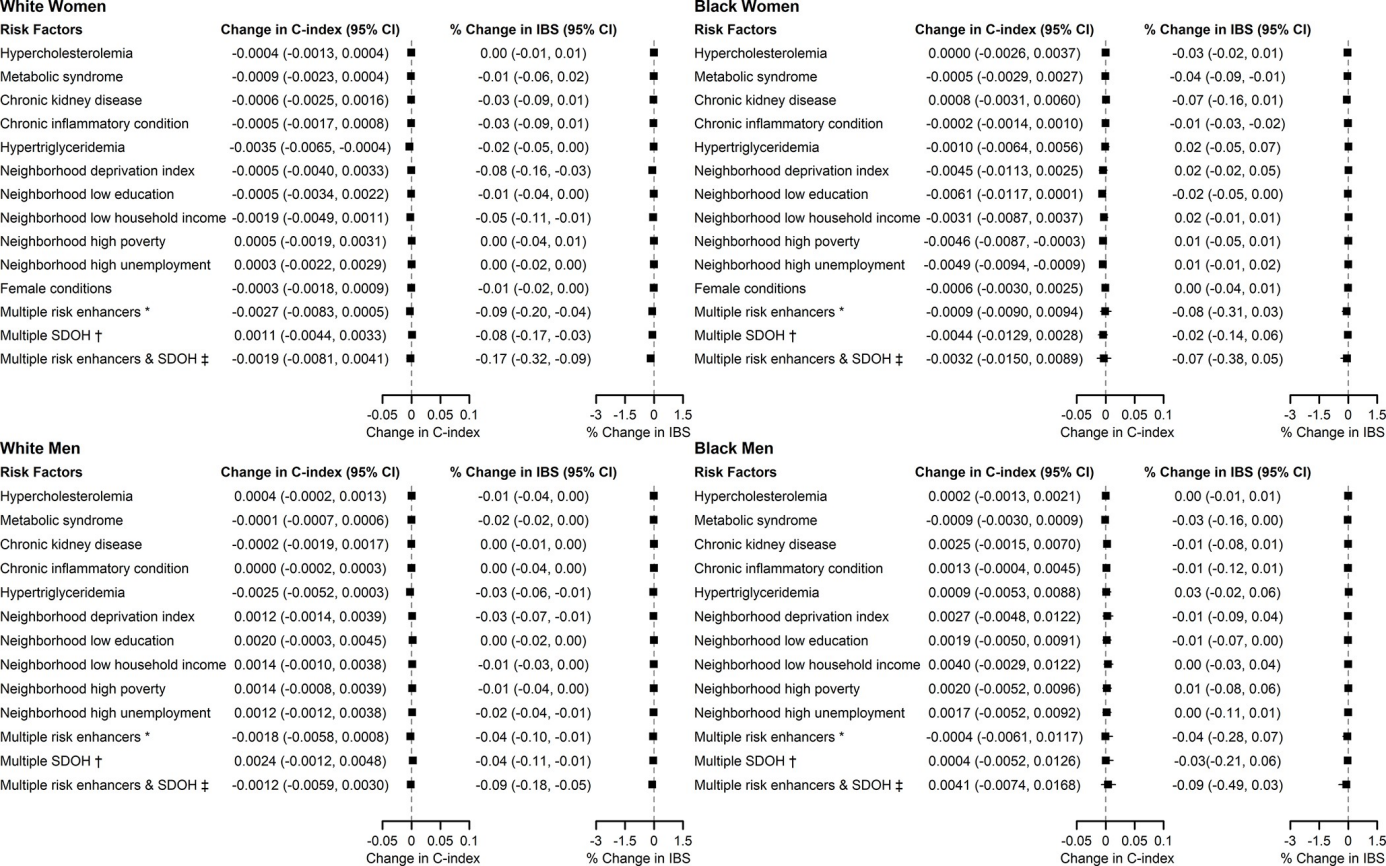
Differences in Harrell’s C-index and percent change in integrated Brier score comparing risk models with and without individual or combination of risk-enhancing factors and social determinants of health, KPSC. Caption: * Multiple risk enhancers include hypercholesterolemia, metabolic syndrome, CKD, chronic inflammatory condition, hypertriglyceridemia, and female conditions (in women only). † Multiple SDOH include neighborhood deprivation index, neighborhood low education, neighborhood low household income, neighborhood high poverty, neighborhood high unemployment. ‡ Multiple risk enhancers & SDOH include all the above. AHRQ: Agency for Healthcare Research and Quality; ASCVD: atherosclerotic cardiovascular disease; KPSC: Kaiser Permanente Southern California; IBS: integrated Brier score.

In KPSC, adding individual risk-enhancing factors to risk prediction models did not improve the C-index compared to models with cPCE only (**Fig 4**). Adding AHRQ neighborhood deprivation index, neighborhood income, and metabolic syndrome to risk prediction led to improvements in IBS in two or more sex/race groups. When adding to the risk prediction models simultaneously all risk-enhancing factors and SDOH that were associated with ASCVD, the C-index was not improved, but IBS improved in White women and White men (% change in IBS was -0.17% in White women and -0.09% in White men). Adding individual risk-enhancing factors or SDOH to the cPCEs also did not improve reclassification. When adding multiple risk-enhancing factors and SDOH simultaneously, there were improvements in event NRI in White women and White men, but not in overall NRI (**S3 Fig in S1 File**).

## Discussion

In this analysis of over 320,000 adults from diverse contemporary populations, the PCEs had moderate discrimination and overestimated the 10-year ASCVD risk even after recalibration. Several risk-enhancing factors recommended in the 2018 AHA/ACC cholesterol guideline were associated with incident ASCVD independent of 10-year risks estimated by the PCEs. Additionally, individual-level and area-level SDOH of education, income, and employment status were also associated with ASCVD independent of the PCEs. While adding individual risk-enhancing factors or SDOH to risk prediction models had limited impact on predictive accuracy, adding multiple risk-enhancing factors and SDOH simultaneously led to modest improvements in model accuracy. These findings suggest that including SDOH and selected risk-enhancing factors may be considered in future development of more refined ASCVD risk assessment tools.

Risk assessment plays a crucial role in primary ASCVD prevention [4]. The 2018 AHA/ACC cholesterol guideline introduced the concept of risk-enhancing factors as a supplement to PCEs-based risk assessment, as studies have shown that the PCEs may overestimate risk in individuals with predicted 10-year risk >10% or higher socioeconomic status, and underestimate risk in certain racial/ethnic groups or those with lower socioeconomic status [2–8]. However, the guideline did not quantify the role of each risk-enhancing factor in risk assessment, leaving it to clinicians to determine whether the presence of certain risk-enhancing factors is significant enough to initiate drug therapy [4, 5]. This uncertainty leads to concerns that some clinicians might ignore these factors (leading to under-treatment) or simply reclassify individuals to a higher risk category if any risk-enhancing factor is present (leading to over-treatment) [30]. To address this knowledge gap, the current study assessed the associations between risk-enhancing factors and ASCVD in diverse contemporary populations, controlling for risks estimated by the PCEs. We found that several risk-enhancing factors recommended by the 2018 ACC/AHA cholesterol guidelines (including family history of premature ASCVD, CKD, metabolic syndrome, hypercholesterolemia, hypertriglyceridemia, and elevated hsCRP) were associated with an increased risk for ASCVD independent of the PCEs. However, adding them individually to risk prediction generally had limited impact on model performance.

SDOH are important risk factors of cardiovascular health, and adverse SDOH are common among US adults [31, 32]. However, SDOH are not included in the PCEs, nor are they considered as risk-enhancing factors in the 2018 AHA/ACC cholesterol guideline. The PCEs have been shown to underestimate ASCVD risk in individuals with a lower socioeconomic status [12, 13]. High-risk, poorer patients are also less likely to receive statins than more affluent patients [33–35]. Consequently, underestimation of risk for those with lower socioeconomic status may lead to under-treatment of high cholesterol, further contributing to the widening gap in cardiovascular outcomes. The current study showed that several SDOH, including both individual-level and area-level low education, low income, and unemployment, were associated with ASCVD risk independent of the PCEs. Further, including SDOH in risk prediction in addition to risk-enhancing factors improved model discrimination and calibration in the pooled cohort. Of note, we found that in KPSC, adding SDOH to the PCEs modestly improved model calibration in White individuals and did not improve model discrimination in any of the four sex/race groups. This might be because only area-level SDOH were available in KPSC, and about 70% of the individuals in KPSC had no adverse area-level SDOH. Additionally, it is important to consider that the effect sizes of SDOH may vary in different cultural contexts. Since the current study focused on populations within the US, the findings may not be directly applicable to populations in other countries with different cultural, social, and healthcare frameworks. Future research should investigate the role of SDOH in diverse international settings to understand better the generalizability and relevance of our findings across various cultural contexts.

The study findings have several clinical implications for ASCVD risk assessment. Although several risk-enhancing factors recommended in the 2018 AHA/ACC cholesterol guidelines were associated with ASCVD risk independently of the PCEs, incorporating them individually into risk prediction models does not meaningfully improve predictive accuracy. However, adding SDOH alongside these factors modestly improved predictive performance, especially among Black individuals. This suggests that future ASCVD risk assessment tools should integrate SDOH to better capture individual risk profiles, particularly among ethnic minority subgroups who likely face a high burden of adverse SDOH and higher ASCVD risk. Such an approach could help mitigate disparities in cardiovascular health outcomes. The recently developed PREVENT equations address some limitations of the PCEs by removing race and including optional predictors such as area-level SDOH or estimated glomerular filtration rate [14]. Nonetheless, the current study indicates that considering both individual- and area-level SDOH, along with other risk-enhancing factors, may further improve ASCVD risk prediction. Future studies should evaluate the benefits of incorporating these additional factors into PREVENT and determine whether a minimal set of risk-enhancing factors and SDOH could achieve similar improvements in model performance, thereby simplifying its implementation.

Main strengths of the current study include the use of data from contemporary epidemiologic cohorts and integrated healthcare system, allowing us to systematically assess the role of a comprehensive list of risk-enhancing factors and SDOH in ASCVD risk assessment in various populations. Having data from both cohort studies and KPSC strengthens the generalizability of the study findings.

This study also has several limitations. First, we were not able to evaluate all risk-enhancing factors that were identified in the 2018 AHA/ACC cholesterol guideline, including lipoprotein(a), apolipoprotein B, and ankle-brachial index, as data were not consistently available across all cohorts. Individual-level SDOH were also not available in KPSC. Second, we only included the selected individual- and area-level SDOH of education, income, and employment status in the current analysis because these are the ones most consistently associated with ASCVD outcomes and are easy to collect and incorporate into risk assessment. Future studies are needed to explore the role of other SDOH (e.g., health literacy, social support, perceived discrimination, residential segregation, racism, etc.) in ASCVD risk prediction. Third, the current study only included non-Hispanic White and Black individuals because the PCEs were originally developed in these two racial/ethnic groups. With about 80% of the study population being non-Hispanic White, the current analysis may have underestimated the impact of adding SDOHs to ASCVD risk assessment. Additionally, there remains uncertainty of the PCEs’ accuracy in Hispanics and Asians, the two fastest growing minority groups in the US [1]. Some evidence suggests that the PCEs generally overestimates risk in Hispanics and Asians, with heterogeneity for Hispanic and Asian subgroups (e.g., overestimates risk in Mexican Americans and East Asians and underestimates risk in South Asians and Puerto Ricans) [1, 36]. Future studies are needed to assess and refine ASCVD risk prediction in diverse Hispanic and Asian populations. Fourth, the cohorts used to develop the PCEs had surveillance components (e.g., review of hospital discharges and obituaries in local newspapers) to detect ASCVD events not reported by participants. However, the REGARDS study did not have this active surveillance and may miss some ASCVD events [37]. Fifth, the current study used a complete-case analysis, excluding individuals with any missing risk factor data required for risk calculation. This approach may introduce potential selection bias, particularly within the electronic health records data of individuals without existing ASCVD, who may have less frequent risk factor assessments. Sixth, because the PCEs’ coefficients were derived externally and may not fit the data as well as the internally derived coefficients for the risk-enhancing factors, the added discriminatory value of the risk-enhancing factors may be overestimated. Seventh, the number of incident ASCVD events is relatively small in the pooled cohort, which may be underpowered to detect significant improvement in model performance for certain risk enhancing factors when stratified by race and sex. However, we found consistent results when repeating the analyses in the much larger KPSC population, providing assurance that the findings are robust and generalizable across diverse populations. Lastly, we reported nominal statistical associations and *P*-values for all analyses as correction for multiple testing may increase the risk of type II errors [38]. We recognize that although this approach minimizes loss of true positive findings, it may also risk identification of false associations and results from the current analysis require confirmation in other studies.

## Conclusions

Several risk-enhancing factors recommended by 2018 AHA/ACC cholesterol guidelines as well as adverse individual- and area-level SDOH of education, income, and employment status were associated with an increased ASCVD risk beyond that predicted by the PCEs. Adding multiple risk-enhancing factors and SDOH to risk prediction modestly improved model accuracy. These findings suggest that SDOH and selected risk-enhancing factors may be considered in the development of future ASCVD risk assessment tools.

## Supporting information

S1 File (DOCX)

## Abbreviations

ACC: American College of Cardiology
AHA: American Heart Association
AHRQ: Agency for Healthcare Research and Quality
ASCVD: Atherosclerotic cardiovascular disease
CHD: coronary heart disease
CKD: chronic kidney disease
IBS: integrated Brier score
KPSC: Kaiser Permanente Southern California
MESA: Multi-Ethnic Study of Atherosclerosis
MI: myocardial infarction
NRI: net reclassification improvement
PAF: population attributable fraction
PCEs: Pooled Cohort Equations
REGARDS: REasons for Geographic And Racial Disparities in Stroke
SDOH: social determinants of health
