# Supplementary material for: Risk-enhancing factors and social determinants of health in risk assessment for atherosclerotic cardiovascular disease
Source: PLoS One. 2024 Oct 25;19(10):e0312756. doi: 10.1371/journal.pone.0312756 (PMC11508156; doi:10.1371/journal.pone.0312756)
Supplement: S1 File — (DOCX) [file pone.0312756.s001.docx]

**Risk-Enhancing Factors and Social Determinants of Health in Risk Assessment for Atherosclerotic Cardiovascular Disease**

**Table of Contents**

**Supplemental Methods**

**S1 Table.** Definition of risk-enhancing factors

**S2 Table.** Parameters of the original and calibrated Pooled Cohort Equations (PCEs) for Estimation of 10-Year Risk for ASCVD

**S3 Table.** Calibration of the original and calibrated PCEs

**S4 Table.** Harrell’s C-index of the original and calibrated PCEs

**S5 Table.** Adjusted hazard ratios of ASCVD associated with each risk-enhancing factor and social determinants of health in KPSC, using both principal and secondary hospital discharge diagnoses to define myocardial infarction events

**S1 Fig.** Study design

**S2 Fig.** Net Reclassification Improvement (NRI) comparing risk models with and without individual or combination of risk-enhancing factors and social determinants of health, pooled cohort

**S3 Fig.** Net Reclassification Improvement (NRI) comparing risk models with and without individual or combination of risk-enhancing factors and social determinants of health, KPSC

**Supplemental References**

**Supplemental Methods**

*Study Cohorts*

The Multi-Ethnic Study of Atherosclerosis (MESA) is a prospective cohort study of 6,814 individuals 45 to 84 years of age free of clinical cardiovascular disease recruited from 6 US communities (Baltimore, Maryland; Chicago, Illinois; Forsyth County, North Carolina; Los Angeles County, California; northern Manhattan, New York; and St. Paul, Minnesota).^1^ Thirty-eight percent of the recruited participants are White, 28% African-American, 22% Hispanic, and 12% Asian, predominantly of Chinese descent. The baseline visit was carried out between 2000-2002, with 5 subsequent in-person follow-up visits.

The REasons for Geographic And Racial Disparities in Stroke (REGARDS) study enrolled a population-based sample of 30,239 Black and White adults aged ≥45 years from the 48 contiguous US states and the District of Columbia.^2^ Sampling aimed to balance on race and sex, with oversampling of the Stroke Buckle (coastal North and South Carolina and Georgia) and the Stroke Belt (the remainder of North and South Carolina and Georgia, and Alabama, Mississippi, Louisiana, Arkansas, and Tennessee), regions with the highest stroke mortality in the US. The baseline visit was carried out between 2003-2007, and participants were contacted every 6 months by telephone to detect potential study end points.

Kaiser Permanente Southern California (KPSC) is an integrated healthcare delivery system currently providing comprehensive medical care services to over 4.8 million members. The KPSC member population is socioeconomically diverse and broadly representative of the racial/ethnic groups living in Southern California.^3^ The KPSC provides medical services to its members through 15 hospitals and more than 200 outpatient facilities. All medical care provided within the KPSC system as well as outside of the system are all captured in comprehensive electronic health records and medical claims. The KPSC cohort included 307,931 non-Hispanic Black and White adults aged between 40-75 years with 12-month continuous membership eligibility prior to September 30, 2009 (index date). We followed the identified KPSC members through 2019 using electronic health records.

| **S1 Table. Definition of risk-enhancing factors** | | |  |
| --- | --- | --- | --- |
|  | **Pooled cohort definitions** | **KPSC definitions** |  |
| **Risk-enhancing factors included in the 2018 AHA/ACC cholesterol guideline** | | |  |
| Family history of premature ASCVD (males, age<55y; females, age<65y) | Self-reported family history of heart attack or stroke in parents, siblings, or children | Not available |  |
| Primary hypercholesterolemia (LDL-C 160-189 mg/dL; non-HDL-C 190-219 mg/dL) | LDL-C 160-189 mg/dL or non-HDL-C 190-219 mg/dL | The most proximal consecutive ≥2 LDL-C 160-189 mg/dL or non-HDL-C 190-219 mg/dL prior to the index date (i.e., 01/01/2009). If there was only one LDL-C or non-HDL-C measure, then one value meeting the criteria. |  |
| Metabolic syndrome (increased waist circumference, elevated triglycerides [>150 mg/dL], elevated BP, elevated glucose, and low HDL [<40 mg/dL in men; <50 in women] are factors; tally of 3 makes the diagnosis) | Meeting three criteria of the following five factors makes the diagnosis: (1) Waist circumference ≥88 cm in women and ≥102 cm in men;  (2) Triglycerides ≥150 mg/dL, or use of lipid-lowering medications (3) SBP ≥130 or DBP ≥80 mm Hg, or use of antihypertensive medications (4) Fasting plasma glucose (FPG) ≥100 mg/dL, or use of antidiabetic medications (5) HDL-C <40 mg/dL in men, or <50 in women, or use of lipid-lowering medications | Meeting three criteria of the following five factors makes the diagnosis (based on lab measures any time prior to the index date and prescription 12 months prior to the index date): (1) BMI ≥30 kg/m^2^; 2) Triglycerides ≥150 mg/dL, or prescription for fibrates (GPI code 3920) or niacin (GPI 771030); 3) SBP ≥130 or DBP ≥80 mm Hg, or prescription for antihypertensive medications; 4) FPG ≥100 mg/dL, or HbA1c ≥5.7%, or 2-hour oral glucose tolerance test (OGTT) blood sugar ≥140 mg/dL, or prescription for metformin (GPI code 2725005000); 5) HDL-C <40 mg/dL in men, or <50 in women, or prescription for fibrates or niacin |  |
| CKD (eGFR 15–59 mL/min/1.73 m^2^ with or without albuminuria; not treated with dialysis or kidney transplantation) | eGFR (calculated by the 2021 CKD-EPI creatinine equation) <60 mL/min/1.73 m^2^ | Two or more consecutive eGFR (calculated by the 2021 CKD-EPI creatinine equation) <60 mL/min/1.73 m^2^ at least 90 days apart |  |
| History of premature menopause (before age 40y) and history of pregnancy-associated conditions that increase later ASCVD risk such as preeclampsia | Female conditions include self-reported history of premature menopause (before age 40y), gestational hypertension, and gestational diabetes | Female conditions include premature menopause (256.31), eclampsia (642.6x), preeclampsia (642.4x, 642.5x, 642.7x) was defined as one or more relevant outpatient/inpatient/emergency department ICD-9 code any time prior to the index date. Gestational diabetes was defined as one or more relevant ICD-9 code (648.x, 790.x) or lab values proposed by International Association of the Diabetes and Pregnancy Study Groups and adopted by the World Health Organization (one or more glucose values from a 75g OGTT equaled or exceeded: FPG 5.1 mmol/L, 1hr PG 10.0 mmol/L, and 2hr PG 8.5 mmol/L) during pregnancy any time prior to the index date. Gestational hypertension was defined as one or more ICD-9 code 642.3x any time prior to the index date. |  |
| Persistently elevated, primary hypertriglyceridemia (≥175 mg/dL) | Triglycerides ≥175 mg/dL | The most proximal consecutive ≥2 triglycerides ≥175 mg/dL prior to the index date. If there was only one triglyceride measure, then one value meeting the criteria. |  |
| Elevated hsCRP (≥2.0 mg/L) | hsCRP ≥2.0 mg/L | The most proximal lab measures of hsCRP ≥2.0 mg/L any time prior to or on the index date. hsCRP was only shown in Tables 1 & 2 and was not analyzed in the rest of the study due to small number. |  |
| Chronic inflammatory conditions such as psoriasis, rheumatoid arthritis, or HIV/AIDS | Not available | (1) Rheumatoid arthritis: a) ≥2 outpatient diagnoses of ICD-9 code of 714.xx and ≥1 outpatient prescription for a disease-modifying antirheumatic drug (DMARD) 12 months prior to the index (Abatacept GPI codes 6640001000; Adalimumab 6627001500; Anakinra 6626001000; Azathioprine 9940601000; Certolizumab pegol 5250502010; Cyclosporine 9940202000; Cyclosporine modified 9940202030; Etanercept 6629003000; Golimumab 6627004000), or b) ≥1 outpatient diagnoses of ICD-9 code of 714.xx or ≥1 prescription 12 months prior to the index date and have additional diagnosis codes or DMARD prescription after the index date so that meets the requirements of ≥2 outpatient diagnoses and ≥1 prescription for DMARD.  (2) Psoriasis or psoriatic arthritis: ≥1 inpatient or outpatient diagnoses of 696.1, 696.0. (3) HIV: identified from the KPSC HIV registry.  (4) Rheumatic heart disease: ≥1 inpatient or outpatient diagnoses of 394.xx, 395.xx, 398.90. (5) Systemic lupus erythematosus: ≥1 inpatient or outpatient diagnoses of 710.0. |  |
| Elevated Lp(a): An Lp(a) ≥50 mg/dL or ≥125 nmol/L constitutes a risk-enhancing factor especially at higher levels of Lp(a) | Not analyzed due to small number of participants with Lp(a) measurements across the cohorts | Not available |  |
| Elevated apoB ≥130 mg/dL | Not analyzed due to small number of participants with ApoB measurements across the cohorts | Not available |  |
| Ankle-brachial index (ABI) <0.9 | Not analyzed due to small number of participants with ABI measurements across the cohorts | Not available |  |
| High-risk race/ethnicities (e.g., South Asian ancestry) | Not applicable | Not applicable |  |
| **Social determinants of health** | | |  |
| Low education (individual-level) | Self-reported less than high school education | Not available |  |
| Low annual household income (individual-level) | Self-reported annual household income <$50,000 | Not available |  |
| Unemployment (individual-level) | Self-reported unemployed (individuals who were retired were not classified as unemployed) | Not available |  |
| Marital status (individual-level) | Self-reported not married or living as married | Not available |  |
| Low education (neighborhood-level) | <75% of residents with high school education in the neighborhood | <75% of residents with high school education in the neighborhood |  |
| Low annual household income (neighborhood-level) | Median annual household income <$50,000 in the neighborhood | Median annual household income <$50,000 in the neighborhood |  |
| Unemployment (neighborhood-level) | ≥10% of residents unemployed in the neighborhood | ≥10% of residents unemployed in the neighborhood |  |
| Poverty (neighborhood-level) | >25% of residents living below the federal poverty line in the neighborhood | >25% of residents living below the federal poverty line in the neighborhood |  |
| Deprived neighborhood (neighborhood-level) | Bottom quintile of AHRQ deprivation index | Bottom quintile of the AHRQ deprivation index |  |

| **S2 Table. Parameters of the original and calibrated Pooled Cohort Equations (PCEs) for Estimation of 10-Year Risk for ASCVD** | | | | |
| --- | --- | --- | --- | --- |
|  | **White woman** | **Black woman** | **White man** | **Black man** |
| **Original PCEs** |  |  |  |  |
| Baseline survival | 0.9665 | 0.9533 | 0.9144 | 0.8954 |
| Mean (Coefficient×Value) | -29.18 | 86.61 | 61.18 | 19.54 |
| **Calibrated PCEs, pooled cohort** | |  |  |  |
| Baseline survival | 0.9529 | 0.9425 | 0.9035 | 0.9125 |
| Mean (Coefficient×Value) | -29.07 | 86.87 | 61.38 | 19.62 |
| **Calibrated PCEs, KPSC** |  |  |  |  |
| Baseline survival | 0.9775 | 0.9744 | 0.9587 | 0.9588 |
| Mean (Coefficient×Value) | -29.72 | 86.05 | 60.80 | 19.24 |
| The estimated 10-year ASCVD risk is calculated as 1 minus the survival rate at 10 years (“Baseline Survival”), raised to the power of the exponent of the “Coefficient×Value” sum minus the race and sex specific overall mean “Coefficient×Value” sum; or, in equation form: | | | | |
| 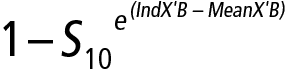   \|  \| \| --- \| |  |  |  |  |

| **S3 Table. Calibration of the original and calibrated PCEs** | | | | | |
| --- | --- | --- | --- | --- | --- |
|  | **Predicted events** | **Predicted risk, %** | **Observed events *** | **Observed risk, %** | **Mean calibration †** |
| **Pooled Cohort** |  |  |  |  |  |
| Original PCEs |  |  |  |  |  |
| White women | 272 | 5.73 (5.57, 5.88) | 223 | 4.71 (4.03, 5.39) | 1.22 |
| Black women | 284 | 8.43 (8.20, 8.65) | 194 | 5.75 (4.85, 6.64) | 1.47 |
| White men | 476 | 12.66 (12.4, 12.91) | 363 | 9.65 (8.61, 10.68) | 1.31 |
| Black men | 256 | 12.91 (12.59, 13.23) | 174 | 8.75 (7.34, 10.15) | 1.48 |
| Calibrated PCEs |  |  |  |  |  |
| White women | 340 | 7.16 (6.97, 7.35) | 223 | 4.71 (4.03, 5.39) | 1.52 |
| Black women | 272 | 8.07 (7.85, 8.28) | 194 | 5.75 (4.85, 6.64) | 1.40 |
| White men | 446 | 11.86 (11.62, 12.10) | 363 | 9.65 (8.61, 10.68) | 1.23 |
| Black men | 200 | 10.10 (9.84, 10.36) | 174 | 8.75 (7.34, 10.15) | 1.15 |
| **KPSC (primary analysis) ‡** |  |  |  |  |  |
| Original PCEs |  |  |  |  |  |
| White women | 5431 | 3.77 (3.75, 3.80) | 3232 | 2.25 (2.16, 2.33) | 1.68 |
| Black women | 1825 | 4.73 (4.68, 4.78) | 989 | 2.56 (2.38, 2.74) | 1.85 |
| White men | 8499 | 8.20 (8.16, 8.24) | 4278 | 4.13 (3.99, 4.27) | 1.99 |
| Black men | 2024 | 9.27 (9.19, 9.35) | 901 | 4.12 (3.82, 4.43) | 2.25 |
| Calibrated PCEs |  |  |  |  |  |
| White women | 5622 | 3.91 (3.88, 3.93) | 3232 | 2.25 (2.16, 2.33) | 1.74 |
| Black women | 1737 | 4.50 (4.45, 4.55) | 989 | 2.56 (2.38, 2.74) | 1.76 |
| White men | 5992 | 5.78 (5.75, 5.81) | 4278 | 4.13 (3.99, 4.27) | 1.40 |
| Black men | 1076 | 4.93 (4.89, 4.97) | 901 | 4.12 (3.82, 4.43) | 1.20 |
| **KPSC (sensitivity analysis) §** | |  |  |  |  |
| Original PCEs |  |  |  |  |  |
| White women | 5431 | 3.77 (3.75, 3.80) | 3615 | 2.51 (2.42, 2.61) | 1.50 |
| Black women | 1825 | 4.73 (4.68, 4.78) | 1092 | 2.83 (2.64, 3.02) | 1.67 |
| White men | 8499 | 8.20 (8.16, 8.24) | 4613 | 4.45 (4.31, 4.60) | 1.84 |
| Black men | 2024 | 9.27 (9.19, 9.35) | 983 | 4.50 (4.18, 4.82) | 2.06 |
| Calibrated PCEs |  |  |  |  |  |
| White women | 5622 | 3.91 (3.88, 3.93) | 3615 | 2.51 (2.42, 2.61) | 1.56 |
| Black women | 1737 | 4.50 (4.45, 4.55) | 1092 | 2.83 (2.64, 3.02) | 1.59 |
| White men | 5992 | 5.78 (5.75, 5.81) | 4613 | 4.45 (4.31, 4.60) | 1.30 |
| Black men | 1076 | 4.93 (4.89, 4.97) | 983 | 4.50 (4.18, 4.82) | 1.09 |
| * The observed number of events at 10 years was adjusted for variable follow-up time using the Kaplan-Meier estimate. † Mean calibration, also known as calibration-in-the-large, was estimated as the ratio of predicted to observed event rates.  ‡ In primary analysis, myocardial infarction was identified by principal hospital discharge diagnoses in KPSC. § In secondary analysis, myocardial infarction was identified by both principal and secondary hospital discharge diagnoses in KPSC. | | | | | |

| **S4 Table. Harrell’s C-index of the original and calibrated PCEs** | | | | | |
| --- | --- | --- | --- | --- | --- |
| **Sex/Race Groups** | **Pooled Cohort** | |  | **KPSC** | |
|  | **Original PCEs** | **Calibrated PCEs** |  | **Original PCEs** | **Calibrated PCEs** |
| White women | 0.73 | 0.73 |  | 0.74 | 0.74 |
| Black women | 0.70 | 0.70 |  | 0.70 | 0.70 |
| White men | 0.69 | 0.69 |  | 0.70 | 0.70 |
| Black men | 0.65 | 0.65 |  | 0.69 | 0.69 |

| **S5 Table. Adjusted hazard ratios of ASCVD associated with each risk-enhancing factor and social determinants of health in KPSC, using both principal and secondary hospital discharge diagnoses to define myocardial infarction events** | | | | |
| --- | --- | --- | --- | --- |
| **Risk Factors** | **White Women** | **Black Women** | **White Men** | **Black Men** |
| Hypercholesterolemia | 1.08 (0.94, 1.24) | 0.68 (0.50, 0.92) | 1.19 (1.05, 1.34) | 1.06 (0.80, 1.42) |
| Metabolic syndrome | 1.24 (1.13, 1.37) | 1.32 (1.12, 1.55) | 1.11 (1.02, 1.20) | 1.10 (0.92, 1.32) |
| Chronic kidney disease | 1.51 (1.33, 1.73) | 1.44 (1.19, 1.75) | 1.24 (1.07, 1.44) | 1.21 (0.98, 1.50) |
| Chronic inflammatory condition | 1.73 (1.45, 2.07) | 1.45 (1.00, 2.12) | 1.05 (0.83, 1.32) | 1.64 (1.00, 2.69) |
| Hypertriglyceridemia | 1.19 (1.06, 1.33) | 1.63 (1.20, 2.19) | 1.18 (1.09, 1.28) | 1.31 (1.04, 1.66) |
| Female condition | 0.53 (0.35, 0.82) | 0.70 (0.45, 1.11) |  |  |
| Neighborhood deprivation index | 1.43 (1.31, 1.56) | 1.18 (1.02, 1.36) | 1.25 (1.15, 1.36) | 1.24 (1.06, 1.44) |
| Neighborhood low education | 1.22 (1.10, 1.36) | 1.24 (1.08, 1.43) | 1.16 (1.05, 1.27) | 1.12 (0.96, 1.31) |
| Neighborhood low household income | 1.26 (1.15, 1.39) | 1.25 (1.08, 1.43) | 1.19 (1.10, 1.30) | 1.24 (1.07, 1.45) |
| Neighborhood high poverty | 1.44 (1.14, 1.81) | 1.03 (0.84, 1.27) | 1.33 (1.08, 1.64) | 1.04 (0.83, 1.31) |
| Neighborhood high unemployment | 1.11 (0.94, 1.31) | 1.06 (0.86, 1.30) | 1.36 (1.19, 1.56) | 1.19 (0.96, 1.48) |

**S1 Fig. Study design**

**
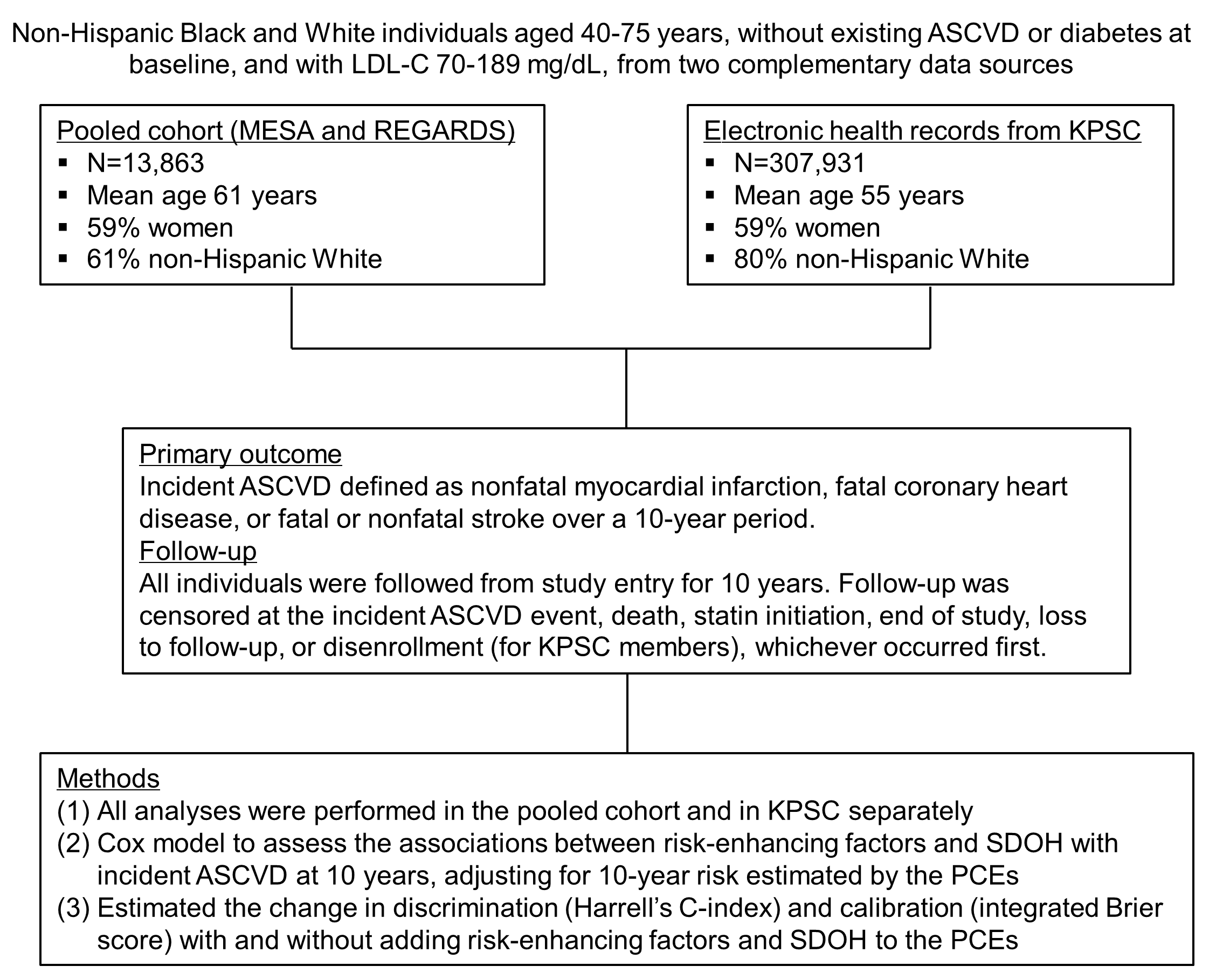
**

**S2 Fig. Net Reclassification Improvement (NRI) comparing risk models with and without individual or combination of risk-enhancing factors and social determinants of health, pooled cohort**


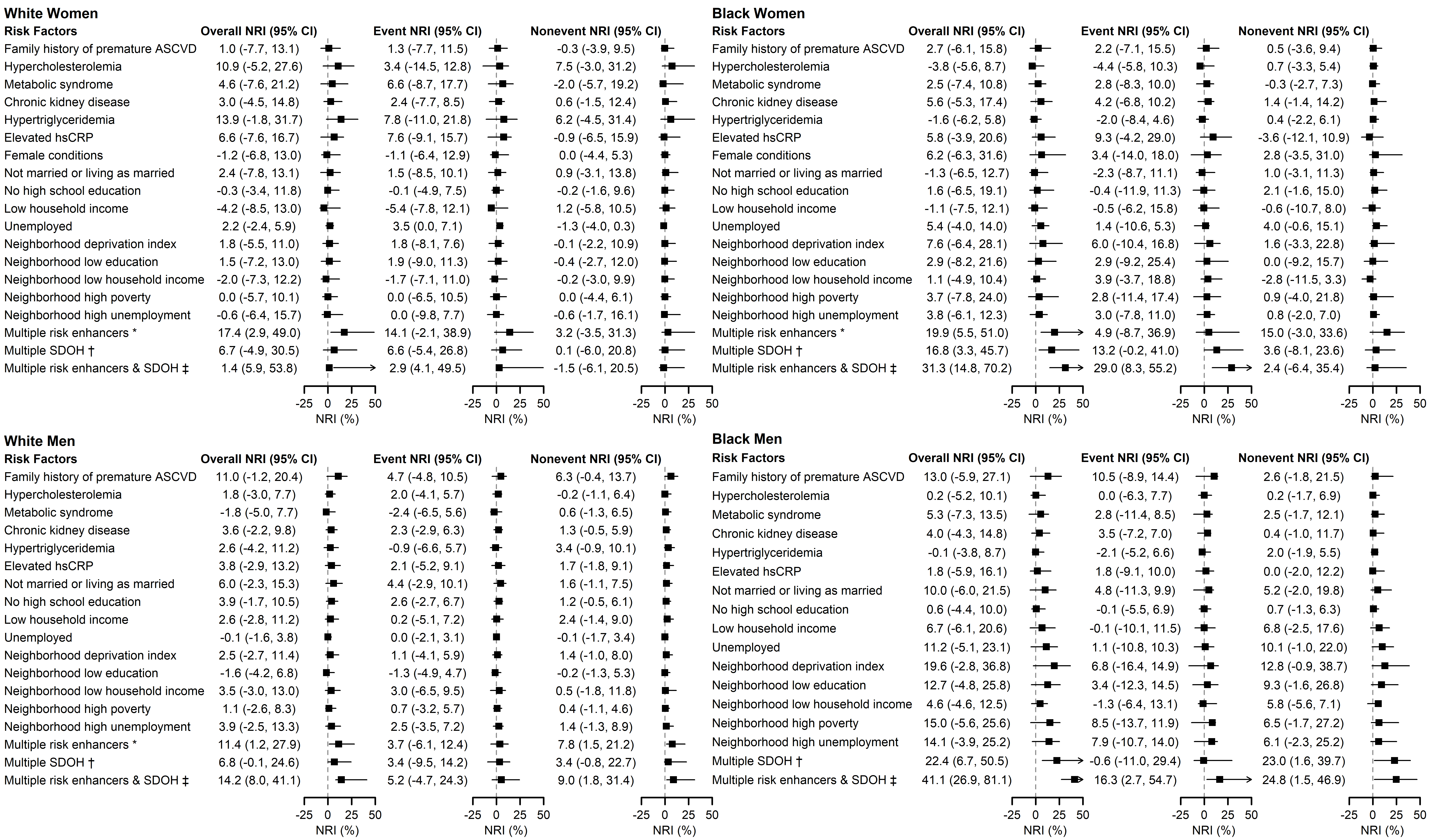


**S3 Fig. Net Reclassification Improvement (NRI) comparing risk models with and without individual or combination of risk-enhancing factors and social determinants of health, KPSC**


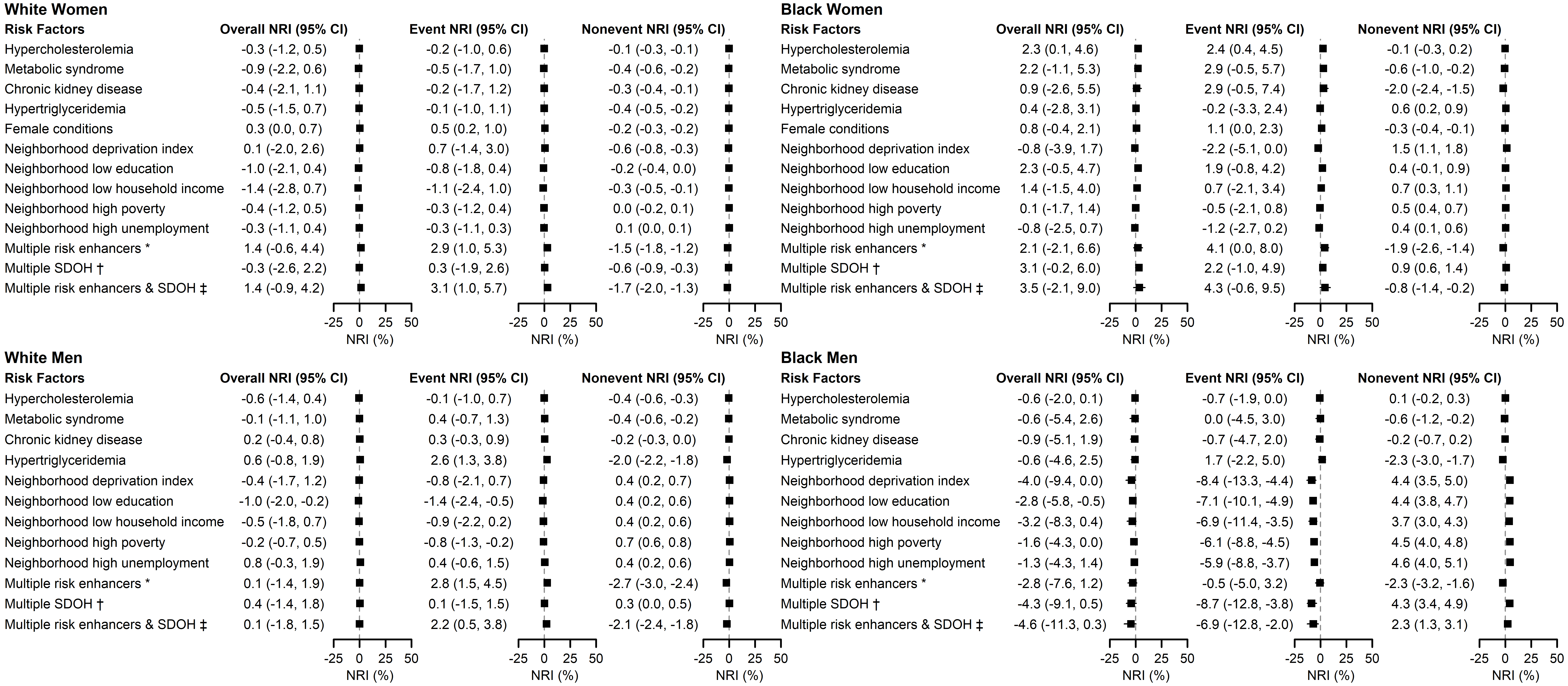


Supplemental References

1. Bild DE, Bluemke DA, Burke GL, et al. Multi-ethnic study of atherosclerosis: objectives and design. *Am J Epidemiol*. Nov 1 2002;156(9):871-81.

2. Howard VJ, Cushman M, Pulley L, et al. The reasons for geographic and racial differences in stroke study: objectives and design. *Neuroepidemiology*. 2005;25(3):135-43. doi:10.1159/000086678

3. Koebnick C, Langer-Gould AM, Gould MK, et al. Sociodemographic characteristics of members of a large, integrated health care system: comparison with US Census Bureau data. *Perm J*. Summer 2012;16(3):37-41. doi:10.7812/TPP/12-031
